# CALENDAR

**DOI:** 10.1054/bjoc.2000.1526

**Published:** 2000-11-22


					11-13 December 2000
Genes & Cancer 2000
UK Molecular Biology & Cancer Network Meeting XVII
University of Warwick, UK
Further information from:
Transcriptional Control, Cytoplasmic Signalling, Nuclear
Structures, Cell Cycle Control, Therapeutics
Dr Stefan Roberts, Department of Biochemistry, University of
Dundee, Dundee, UK. Tel: + 44 (0) 1382 344248;
Fax: + 44 (0) 1382 345783; E-mail: s.g.e.roberts@dundee.ac.uk;
Website: www.icr.uk/ukmbcn/info.htm
5-7 February 2001
3rd UICC Cancer Management Meeting - Quality
Cancer Care in the New Millennium
Republic of Singapore
Further information from:
Secretariat, The National Cancer Centre (Singapore), 11 Hospital
Drive Level 4, Singapore 169610, Republic of Singapore.
Tel: + 65 436 8294/8283/8186; Fax: + 65 225 7559; E-mail:
3rduice@nccs.com.sg; Website: www.nccs.com.sg
14-17 February 2001
International Conference on Dietary Factors: Cancer
Causes & Prevention
Vienna, Austria
The following main areas will be covered:
1. Chemical analytical data on hazardous and protective
constituents of foods
2. Mutagens and antimutagens in the human diet
3. Post initiation effects of dietary factors
4. Human biomonitoring and risk assessment
5. Epidemiology, trends in nutrition and dietary recommendations
Further information from:
Johanna B. Baukovits, Institute for Cancer Research,
Borschkegasse 8a, A-1090 Vienna, Austria. Fax: + 43 142 77 96
51; E-mail: walter.paukovits@univie.ac.at
1-3 March 2001
From Tradition to Modernity: Asian Therapies for
Cancer - A First International Conference
The Roosevelt Hotel, New York City, USA
Further information from:
Pro-Cultura. Tel: 914 741 2781; Fax: 914 741 1932;
E-mail: mail@procultura.org; Website: http://www.procultura.org
7-10 March 2001
4th International Symposium on Leukemia and
Lymphoma - molecular pharmacology and new
treatment modalities
Amsterdam, The Netherlands
Chairman: G.J.L. Kaspers, R. Pieters, P. Sonneveld and A.J.P.
Veerman
Abstract deadline: 1 December 2000
Further information from:
VU Conference Service, De Boelelaan 1105, NL-1081 HV
Amsterdam, The Netherlands. Tel: + 31 (0) 20 444 5790;
Fax: + 31 (0) 20 444 5825;
E-mail: vu_conference@dienst.vu.nl
21-23 May 2001
UK Radiological Congress 2001 (UKRC 2001)
(Incorporating IOS & MED X RAY(R)
)
Wembley Conference & Exhibition Centre, London, UK
Further information from:
UKRC Secretariat, PO Box 2895, London W1A 5RS, UK.
Tel: + 44 (0) 20 7307 1410/20; Fax: + 44 (0) 20 7307 1414;
E-mail: ukrc@dial.pipex.com;
Website: www.ukrc.org.uk
4-7 June 2001
16th Annual Offering of Critical lssues in Tumor
Microcirculation, Angiogenesis and Metastasis:
Biological Significance and Clinical Relevance
A Continuing Education Course of Harvard Medical
School and Massachusetts General Hospital
Boston, MA, USA
Further information from:
Website: http://steele.mgh.harvard.edu
15-16 March 2002
4th International Conference on the Adjuvant Therapy
of Malignant Melanoma
The Royal College of Physicians, London
Further information from:
Conference Secretariat, CCI Ltd, 2 Palmerston Court, Palmerston
Way, London SW8 4AJ, UK. Tel: + 44 (0) 20 7720 0600; Fax:
+ 44 (0) 20 7720 7177; E-mail: melanoma@confcomm.co.uk;
Website: www.confcomm.co.uk/Melanoma
CALENDAR
1582
British Journal of Cancer (2000) 83(11), 1582
(c) 2000 Cancer Research Campaign
doi: 10.1054/ bjoc.2000.1526, available online at http://www.idealibrary.com on http://www.bjcancer.com

				

